# Abnormal neurobehaviour and impaired memory function as a consequence of *Toxocara canis*- as well as *Toxocara cati*-induced neurotoxocarosis

**DOI:** 10.1371/journal.pntd.0005594

**Published:** 2017-05-08

**Authors:** Elisabeth Janecek, Patrick Waindok, Marion Bankstahl, Christina Strube

**Affiliations:** 1 Institute for Parasitology, Centre for Infection Medicine, University of Veterinary Medicine Hannover, Hanover, Germany; 2 Department of Pharmacology, Toxicology, and Pharmacy, University of Veterinary Medicine Hannover, Hanover, Germany; 3 Center for Systems Neuroscience, Hanover, Germany; George Washington University, UNITED STATES

## Abstract

**Background:**

Neuroinvasive larvae of the worldwide occurring zoonotic roundworms *Toxocara canis* and *T*. *cati* may induce neurotoxocarosis (NT) in humans, provoking a variety of symptoms including cognitive deficits as well as neurological dysfunctions. An association with neuropsychological disorders has been discussed. Similar symptoms have been described in *T*. *canis*-infected mice, whereas data on *T*. *cati*-induced NT are rare. Therefore, it was aimed to obtain insights into the impact on neurobehaviour as well as progression of neurological symptoms and behavioural alterations during the course of NT directly comparing *T*. *canis*- and *T*. *cati*-infected mice as models for human NT.

**Methodology/Principal findings:**

C57BL/6 mice were orally infected with 2000 embryonated *T*. *canis* or *T*. *cati* eggs, respectively, the control group received tap water. Mice were screened weekly for neurobehavioural alterations and memory function starting one day prior infection until 97 days post infection (pi; *T*. *canis*-infection) and day 118 pi (*T*. *cati*-infection, uninfected control). Mostly motoric and neurological parameters were affected in *T*. *canis*-infected mice starting day 20 pi with severe progression accompanied by stereotypical circling. In contrast, *T*. *cati*-infected mice mostly showed reduced response to sudden sound stimulus (indicator for excitability) and flight behaviour starting day 6 pi. Interestingly, enhanced grooming behaviour was observed exclusively in *T*. *cati*-infected mice, indicating a possible role of neurotransmitter dysregulation. Reduced exploratory behaviour and memory impairment was observed in both infection groups with delayed onset and less severe progression in *T*. *cati*- compared to *T*. *canis*-infected mice.

**Conclusions/Significance:**

Results highlight the need to consider *T*. *cati* beside *T*. *canis* as causative agent of human NT. Findings provide valuable hints towards differences in key regulatory mechanisms during *T*. *canis*- and *T*. *cati*-induced NT, contributing to a comprehensive picture and consequently a broader understanding of NT, which will aid in developing strategies towards prevention in addition to novel diagnostic and therapeutic approaches.

## Author summary

The worldwide occurring zoonotic roundworms *Toxocara canis* and *T*. *cati* may cause the so-called neurotoxocarosis (NT) in humans, possibly resulting in a variety of behavioural alterations. Comparable alterations to those in humans have been described in *T*. *canis*-infected mice, however, reports on *T*. *cati*-induced NT are rare. Therefore, the main causative agent of human NT remains unknown. For a better understanding and more detailed characterization of *T*. *canis*- and *T*. *cati*-induced NT, infected and uninfected mice were exposed to several examinations regarding behavioural alterations and cognitive dysfunctions. Even though both roundworm species share many characteristics, pathogenicity as well as larval migration in the animal model has been described as quite different. The current study additionally highlights differences between behavioural alterations in infected mice. *T*. *canis*-infection mostly provoked neurological alterations, whereas *T*. *cati*-infection resulted in reduced excitability and flight-related parameters. Exploratory behaviour was reduced in both infection groups in addition to a negative effect of infection on memory function. The presented work provides a valuable basis for further studies and highlights the need for further investigations concerning consequences and differences between *T*. *canis*- and *T*. *cati*-induced NT.

## Introduction

Toxocarosis, caused by infective third stage larvae (L3) of the worldwide occurring roundworms of dogs and cats, *Toxocara canis* and *Toxocara cati*, respectively, is one of the most common parasitic infections in humans. Nevertheless, the global importance of the zoonotic helminth is considered to be underestimated [1,2]. Consequently, toxocarosis has been listed as one of the five most neglected parasitic infections in humans targeted as priority for public health action by the Centers for Disease Control and Prevention (CDC) [3]. Infections are most frequently acquired by ingestion of embryonated infective eggs from the environment with poor sanitation as well as poor hygiene standards favouring the risk for infection. Thus, mostly children and people living in poverty are affected. Favorable environmental conditions for development of *Toxocara* eggs as well as transmission in tropical climates result in comparably high contamination as well as infection rates in these countries. Exemplarily, seroprevalences up to 92.8% in La Réunion, France, were reported, demonstrating frequent exposure of humans to *Toxocara*. Considerably lower seroprevalences are detected in temperate regions as for example 2.4% in Denmark [4].

Human toxocarosis is associated with several forms of disease with varying symptoms [1]. One of these forms, neurotoxocarosis (NT), is induced by neuroinvasive L3 and may result in pathological presentations such as meningitis, encephalitis and myelitis. Also, cerebral lesions have been predominantly observed in the white matter in addition to occlusion of cerebral arterial vessels [5–7]. This CNS involvement may result in symptoms such as motor impairments, neuropsychological disturbances like dementia or depression as well as in behavioural alterations [7–13]. Previous studies also correlated *Toxocara*-seroprevalence with reduced cognitive development and function in children and young adults [14–17]. Sporadically, an implication in neurodegenerative diseases like multiple sclerosis and Alzheimer’s disease has been suggested [18,19].

Similar observations on clinical symptoms and neurological alterations have been made in mice which are therefore considered suitable model hosts for human NT. However, an extensive neurotropism with persistence in the brain has solely been described for *T*. *canis* larvae. Contrary, *T*. *cati* larval migration to neuronal tissue is less frequently observed and larvae mainly accumulate in skeletal muscle tissue [20]. Both species cause structural damage such as malacia and demyelination, which is more severe in *T*. *canis*- than in *T*. *cati*-infected mice [21–24]. Additionally, previous studies demonstrated partially severe neurological symptoms as well as behavioural alterations and memory impairment in *T*. *canis*-infected mice [25–27], whereas behavioural data about *T*. *cati*-induced NT is scarce. However, previous studies indicate earlier onset accompanied by more severe neurological symptoms in *T*. *canis*- compared to *T*. *cati*-infected mice [24]. Nevertheless, dysregulations of genes associated with the biological modules “behaviour and taxis” as well as “sensory perception/neurological system process” have been described in *T*. *canis*- as well as in *T*. *cati*-infected mouse brains [28], indicating potential behavioural alterations caused by either *Toxocara* species. Based on the affinity to the CNS as well as observed symptoms, *T*. *canis* is considered the causative agent of most human cases of NT. Nevertheless, *T*. *cati* has previously been associated with human cases of NT and the infection risk should not be underestimated [12,29] as environmental contamination with *T*. *cati* eggs is considered to be high due to the uncontrolled defecation behaviour of cats [30,31].

Due to the high infection risk in combination with the impact of *Toxocara-*infection on neurological and behavioural processes, it was aimed to assess the impact on neurobehaviour as well as the progression of neurological symptoms during the course of NT directly comparing *T*. *canis*- and *T*. *cati*-infected mice as models for human NT. Obtained data will aid in further characterization of possible influences of infection on the paratenic host and give a closer insight into the pathogenesis of NT as data about neurological disorders and cognitive deficits due to toxocarosis are still rare [32].

## Materials and methods

### Ethic statement

Animal experiments were performed in accordance with the German Animal Welfare act in addition to national and international guidelines for animal welfare. Experiments were permitted by the ethics commission of the Institutional Animal Care and Use Committee (IACUC) of the German Lower Saxony State Office for Consumer Protection and Food Safety (Niedersächsisches Landesamt für Verbraucherschutz und Lebensmittelsicherheit) under reference numbers 33.9-42502-05-01A038 and 33.12-42502-04-15/1869.

### Biological material and experimental infection

*T*. *canis* and *T*. *cati* eggs were obtained from faeces of experimentally infected dogs and cats, respectively, kept at the Institute for Parasitology, University of Veterinary Medicine Hannover, for continuous maintenance of *Toxocara* spp. strains (reference number 33.9-42502-05-01A038). Eggs were purified by a combined sedimentation/flotation technique and allowed to embryonate for 4–5 weeks at 25°C with subsequent storage in tap water at 4°C until use.

C57BL/6JRccHsd (Harlan Laboratories, Horst, Netherlands) female mice were obtained at approximately 4 weeks of age. Mice were allowed 5 days of acclimatization followed by 8 days of training for behavioural assessments prior infection (see sections below). At 6 weeks of age, a total of 16 mice were orally infected with 2000 embryonated *T*. *canis* as well as 7 mice with 2000 *T*. *cati* eggs in a total volume of 0.5 ml tap water. The control group (n = 7) received tap water only. Maintenance of mice included a 12/12 hours dark/light cycle, standard rodent diet *ad libitum* as well as daily assessment of physical condition. Behavioural assessments were conducted weekly starting one day prior infection (-1 dpi) until 97 days post infection (dpi) for *T*. *canis*-infected mice or until day 118 pi for *T*. *cati*-infected and control mice (reference number 33.12-42502-04-15/1869). Differences in duration of trials are based on delayed clinical symptoms in *T*. *cati*-infected mice as well as on severe progression and resulting ethical concerns in *T*. *canis*-infected mice [24]. Brain sections of all infection groups were stained exemplary with haematoxylin and eosin following termination of respective trials to demonstrate brain infection and resulting structural damage in *Toxocara*-infected mice.

### Physiological status and neurobehavioural phenotyping

Mice were tested weekly regarding their physiological status as well as neurobehavioural alterations based on a modified Irwin Screen protocol [33]. Acclimatization prior examination was allowed for 30 min in a designated testing room separate from the maintenance room. Assessment was conducted in two phases: The first phase included a total of 17 general observations regarding appearance, health and motoric function. Briefly, body weight of mice was recorded as well as their responsiveness to being lifted up by the tail. Additionally, tail suspension and arousal upon transferring mice from the scale to the observation cage was evaluated. Within the cage, body position, tail elevation, respiratory rate, skin colour, coat appearance, eyes (exophthalmos, ptosis as well as lacrimation) and salivation were recorded as parameters for physiological status. As indicators for neurological and motoric dysfunctions, pelvic elevation, leg position and tumbling motion of mice were recorded in addition to startle response in combination with the ability to hear by evaluating responsiveness/excitability of mice to a sudden sound stimulus. The second test phase was conducted after the assessment of general activity (see section below) and included handling of mice which comprised balance in a moving cage, inquisitiveness upon a presented object, escaping upon gentle touch, vibrissae reflex, exploration and placing behaviour, forelimb placing reflex, “vertical screen test” (ability to hold on to an upside down screen), righting reflex and general handling behaviour. Body temperature of mice was measured at the end of assessments. Regular behaviour was scored with “2”, deviating behaviour was scored higher or lower according to a defined scoring system. Deviation of the group mean from the standard score was calculated as percentage deviation from the standard score, if more than 25% of mice showed the respective alteration. Detailed description of the individual testing parameters as well as the corresponding scoring system is provided in S1 Appendix. The experimental cage was cleaned with 0.1% acetic acid following each individual mouse assessment.

### Assessment of general activity

General activity was assessed by observing activity of mice in the experimental cage for three minutes, recording the activity and position every 10 sec. Position was determined by subdividing the cage into 12 squares along the wall (7.7 cm x 7.8 cm) as well as the centre (7.7 cm x 23.4 cm). Activity was evaluated by determining the speed of movement (inability to move, slow, normal and rapid) as well as the following parameters: walking, grooming, sitting and sitting in a corner, sniffing, rearing, rearing against the wall as well as urinating/defecation. Besides normal activities, stereotypic behaviour like circling or head flicking as well as seizures were noted. Detailed information about assessed activities is listed in S2 Appendix.

### Assessment of sensorimotor function

Functional deficits after *Toxocara*-infection were evaluated based on the approach described by Bouet et al. [34]. An approximately 0.2 x 0.2 cm piece of tape was applied to each forepaw of mice with subsequent immediate release in the experimental cage. Time was recorded upon first contact for left and right paws separately as well as upon removal of the tape for each paw. If 150 sec were exceeded without removal, mice were classified unable to sense or remove the piece of tape and recorded with 150 sec for subsequent analyses. The experimental cage was cleaned with 0.1% acetic acid following each individual mouse assessment.

### Assessment of memory function

To assess the effect of *Toxocara*-infection on memory function as well as potential differences between *T*. *canis*- and *T*. *cati*-induced NT, mice were conditioned in a classic maze to find a food reward starting 8 days prior infection (initial training). In addition to the weekly experimental evaluation, mice were allowed to find the food reward once a week (continuous training). Mice were deprived of food for 2 hours before the maze test, which was recorded by camera and subsequently analysed in terms of duration until discovery of food reward and entries into dead-end arms. Changes of direction were also considered as dead-end entries. Mice were allowed three trials during experimental procedure with individual mean times used for final analyses. Mice were removed from the maze when 360 sec without completing the task were exceeded which was used in analyses as the maximum duration. The maze measured 36 cm x 28 cm with a height of 15 cm and included a series of vertical walls without ceiling. Vertical walls were removable and the floor of the maze was covered with plastic coated covering paper, which was disposed and replaced after each individual run. Maze structure is provided in S1 Fig.

### Statistical analyses

Obtained data was tested for statistical differences using an unpaired *t*-test with Welch's correction comparing the respective infection group to the control group each experimental day. If datasets did not pass normality tests, Mann-Whitney *U*-test was applied. Statistical analyses were conducted with GraphPad Prism^TM^ software (version 6.03). The level of significance was set at α = 5%.

## Results

### General health observations

Severe progression of *T*. *canis*-infection was observed resulting in a total of 3 mice being euthanized prior termination of experiments due to poor general health. Additionally, two mice were euthanized within the *T*. *cati*-infection group before completing the assessments during the course of infection. Therefore, experiments were conducted with the following numbers of mice: control mice: n = 7 at day -1 to 118 pi; *T*. *canis*-infected mice: n = 16 at days -1 to 55 pi, n = 15 at day 62 pi, n = 14 at days 69 to 90 pi and n = 13 at day 97 pi; *T*. *cati*-infected mice: n = 7 mice at day -1 pi, n = 6 at days 6 to 111 pi and n = 5 at day 118 pi.

**Fig 1:**
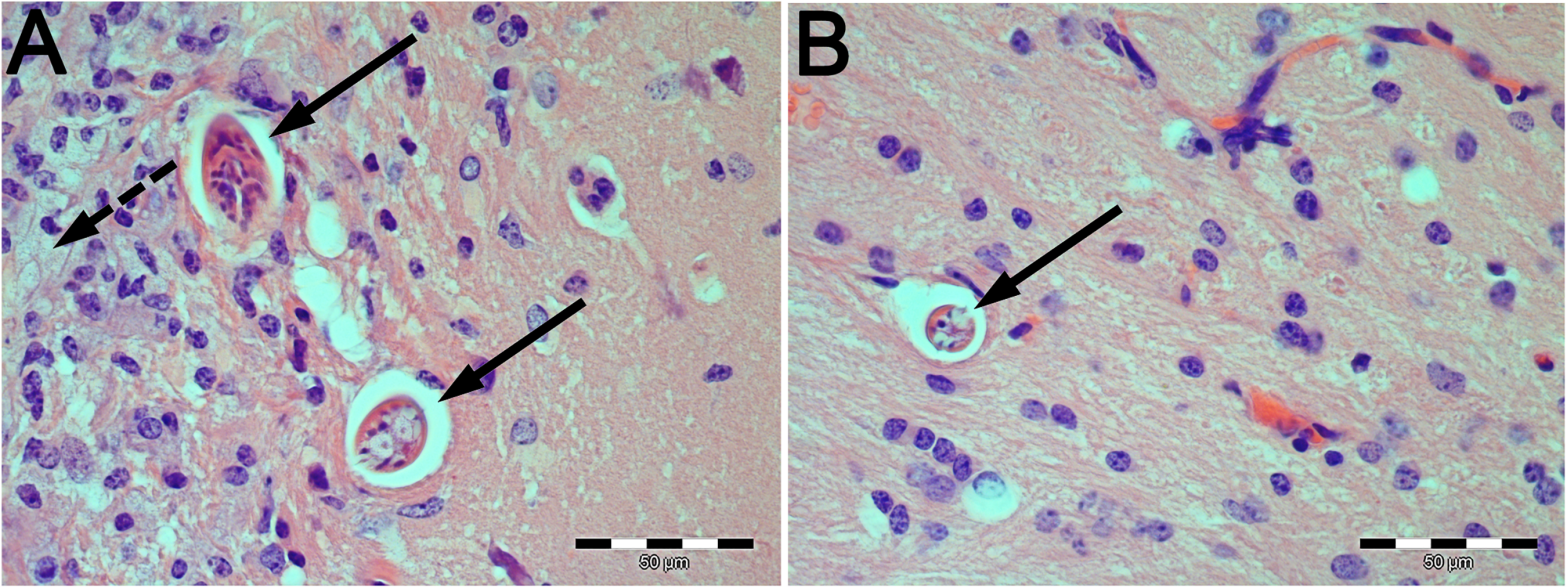
Histological sections of *Toxocara*-infected mouse brains. (A) *T*. *canis*- and (B) *T*. *cati*-infected mouse brain section. Solid arrows indicate *Toxocara* larvae and dashed arrow indicates accumulation of gitter cells.

Histological stains revealed cross sections of *Toxocara* larvae as well as vacuolization, meningitis, perivascular cuffs and haemosiderophages in *T*. *canis*- as well as in *T*. *cati*-infected mouse brains. Additionally, gitter cells were detected as a result of *T*. *canis*-induced NT. Exemplary sections are provided in Fig 1.

Regarding the body weight, *T*. *canis*-infected mice showed significantly higher body weights on day -1 and continuously lower weights than control mice starting day 27 pi. *T*. *cati*-infected mice also showed a significantly higher body weight day -1 and 13 pi, however, continuously lower body weights were not observed until day 62 pi. General progression of mean body weights of mice during the course of infection is provided in S2 Fig.

Body temperature of uninfected control mice was consistent throughout the experiment in contrast to *T*. *canis*-infected mice, which showed significantly lower body temperature starting day 69 pi. *T*. *cati*-infected mice partially showed significantly higher body temperatures during the course of infection, except for significantly lower body temperatures than control mice on day 118 pi. Determined body temperatures are mostly considered within physiological range with *Toxocara*-infected mice being at the upper or lower limit. Mean body temperatures of all experimental groups are provided in S2 Fig.

### Neurobehavioural phenotyping

**Fig 2:**
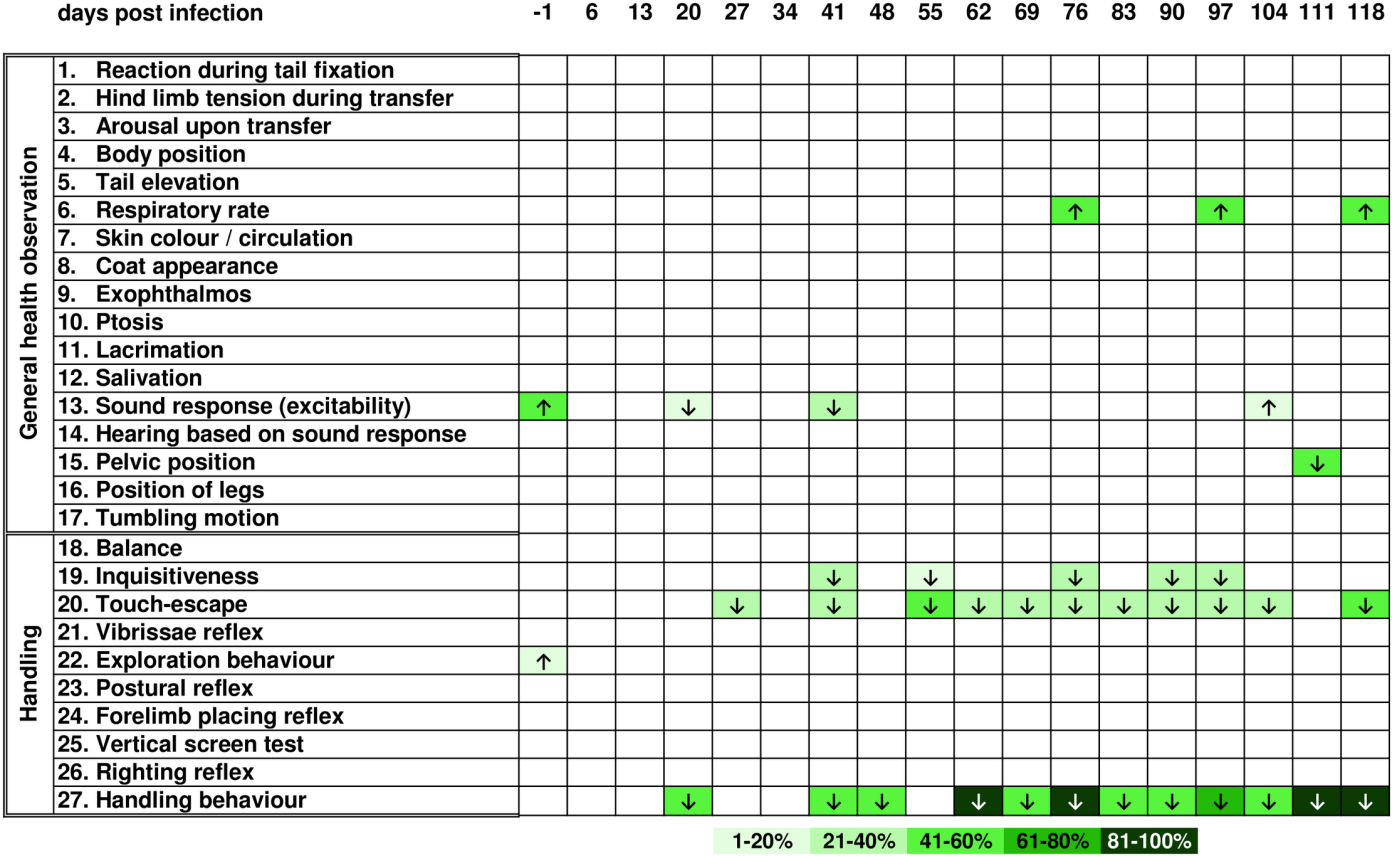
Altered health and behavioural parameters during the course of neurotoxocarosis in uninfected control mice. Arrows indicate if the parameter was increased or decreased in at least 25% of mice per group. Colours specify the severity of the presented alteration based on the group mean difference from the standard score (= 2), whereby colour categories represent the percentage deviation from the standard score. If less than 25% of mice per group showed behavioural alterations, no alteration was indicated.

**Fig 3:**
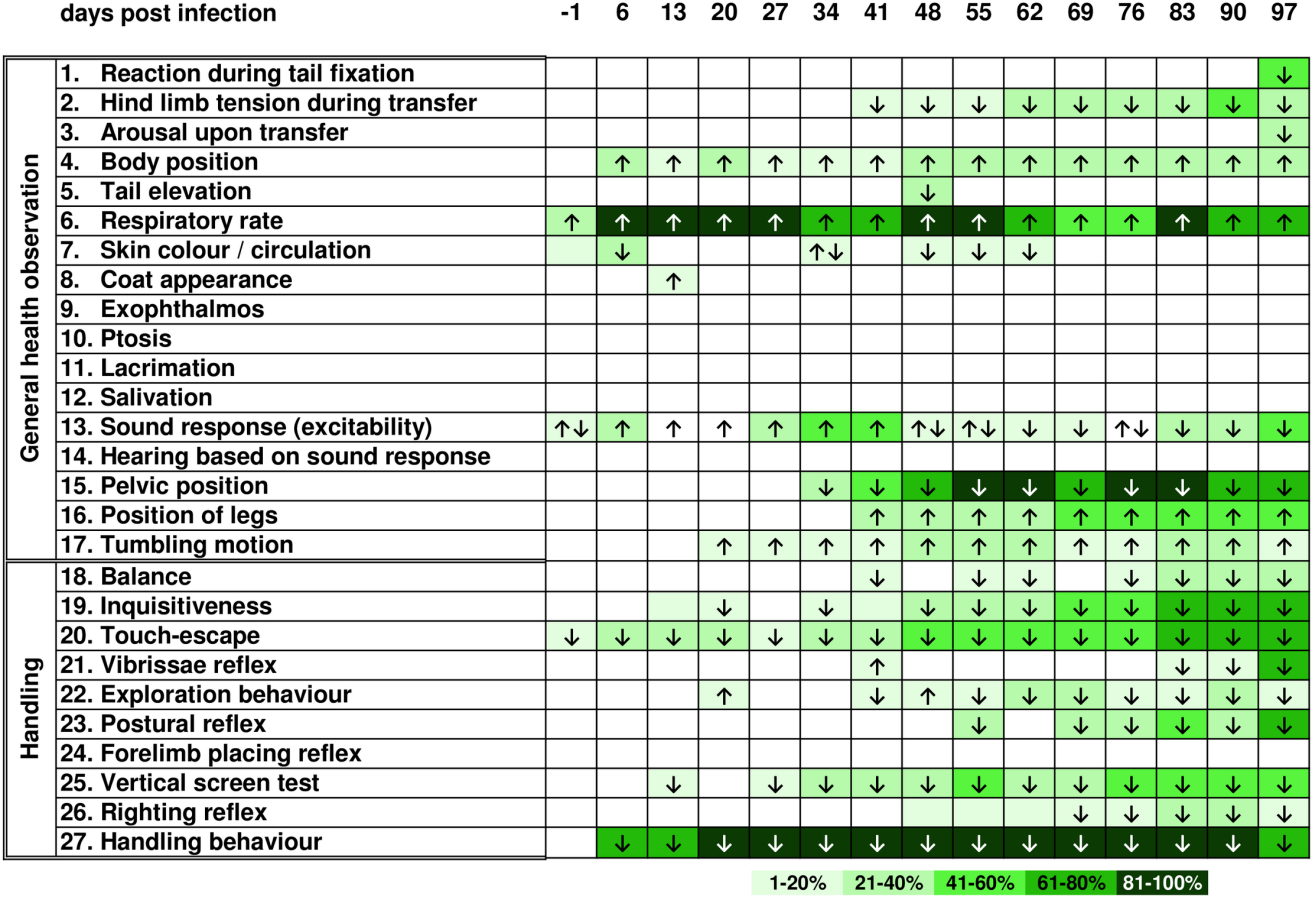
Altered health and behavioural parameters during the course of *Toxocara canis*-induced neurotoxocarosis. Arrows indicate if the parameter was increased or decreased in at least 25% of mice per group. Colours specify the severity of the presented alteration based on the group mean difference from the standard score (= 2), whereby colour categories represent the percentage deviation from the standard score. If less than 25% of mice per group showed behavioural alterations, no alteration was indicated.

**Fig 4:**
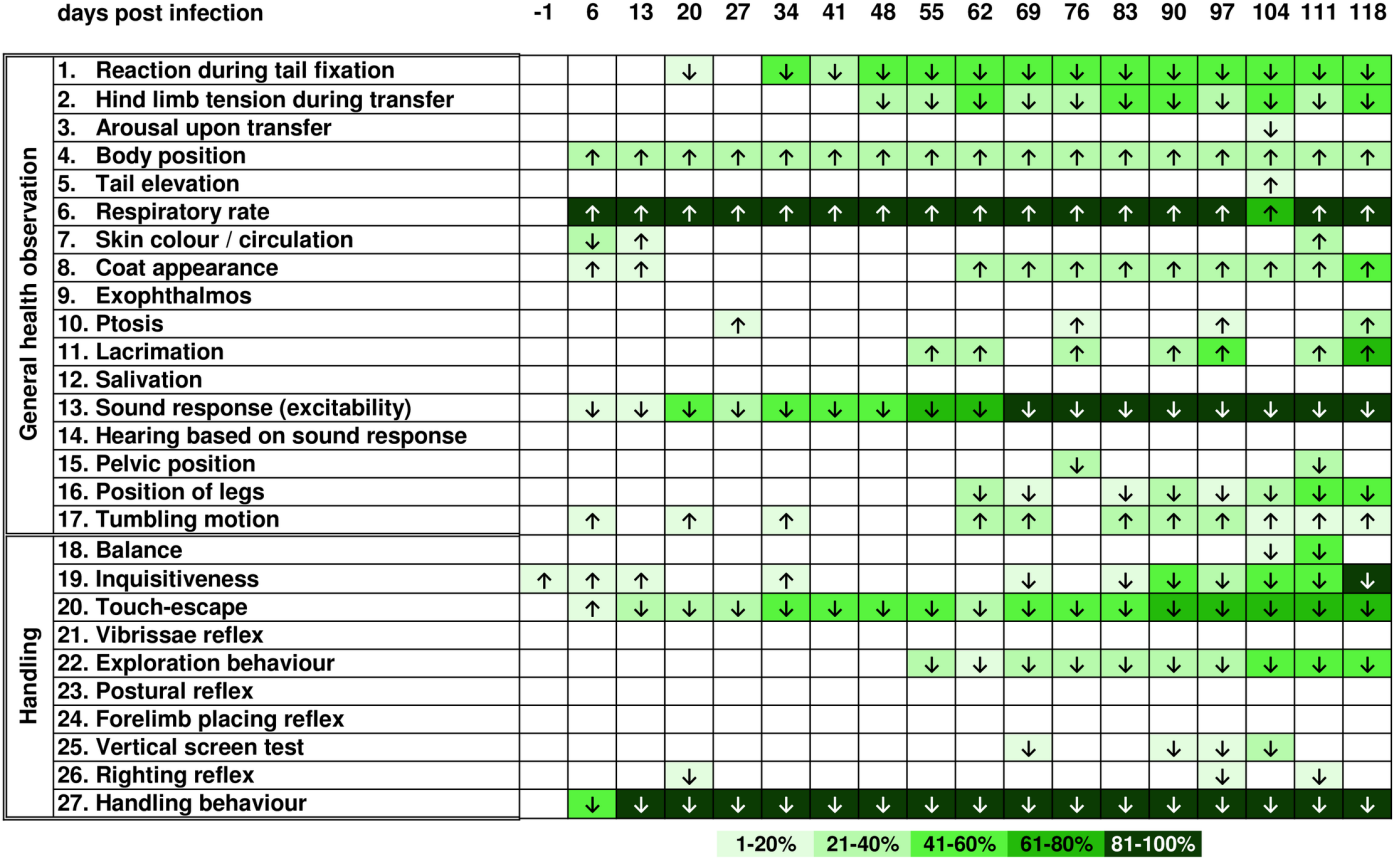
Altered health and behavioural parameters during the course of *Toxocara cati*-induced neurotoxocarosis. Arrows indicate if the parameter was increased or decreased in at least 25% of mice per group. Colours specify the severity of the presented alteration based on the group mean difference from the standard score (= 2), whereby colour categories represent the percentage deviation from the standard score. If less than 25% of mice per group showed behavioural alterations, no alteration was indicated.

Overall, differences in general health as well as behaviour compared to uninfected controls (Fig 2) were observed for *T*. *canis*- and *T*. *cati*-infected mice. Both infection groups showed an increased respiratory rate [parameter (p) 6] as well as bend body position (p 4) with higher intensity in *T*. *cati*-infected mice during the course of infection. In *T*. *canis*-infected mice, neurological and motoric dysfunctions such as tumbling motions (Fig 3; p 17) and ataxia (p 15, 16) in addition to disturbed balance (p 18) and impaired righting reflexes (p 26) were observed. Day 27 pi, *T*. *canis*-infected mice started to show the inability to hold on to an inverted screen during the vertical screen test (p 25) and presented as less inquisitive (p 19) and explorative (p 22) during the chronic phase of infection (starting day 48 pi). Less inquisitive (p 19) and explorative (p 22) behaviour was also observed in *T*. *cati*-infected mice (Fig 4); however, the most striking feature were reduced reactions during fear- and flight-related assessments instead of neurological alterations. Particularly, assessments of excitability (p 1, 13) as well as escape parameters (p 1, 20) were affected. *T*. *cati*-infected mice additionally presented with ruffled as well as partially dirty coats (p 8) starting day 62 pi. Even though all three experimental groups showed habituation to being handled (p 27) during the course of infection as well as a reduced flight reaction when being touched with an object (p 20), these alterations of behaviour were observed with higher intensity and more frequently in *Toxocara*-infected mice. A detailed schematic overview of altered health as well as behavioural parameters is presented in Figs 2–4.

### Assessment of general activity

**Fig 5:**
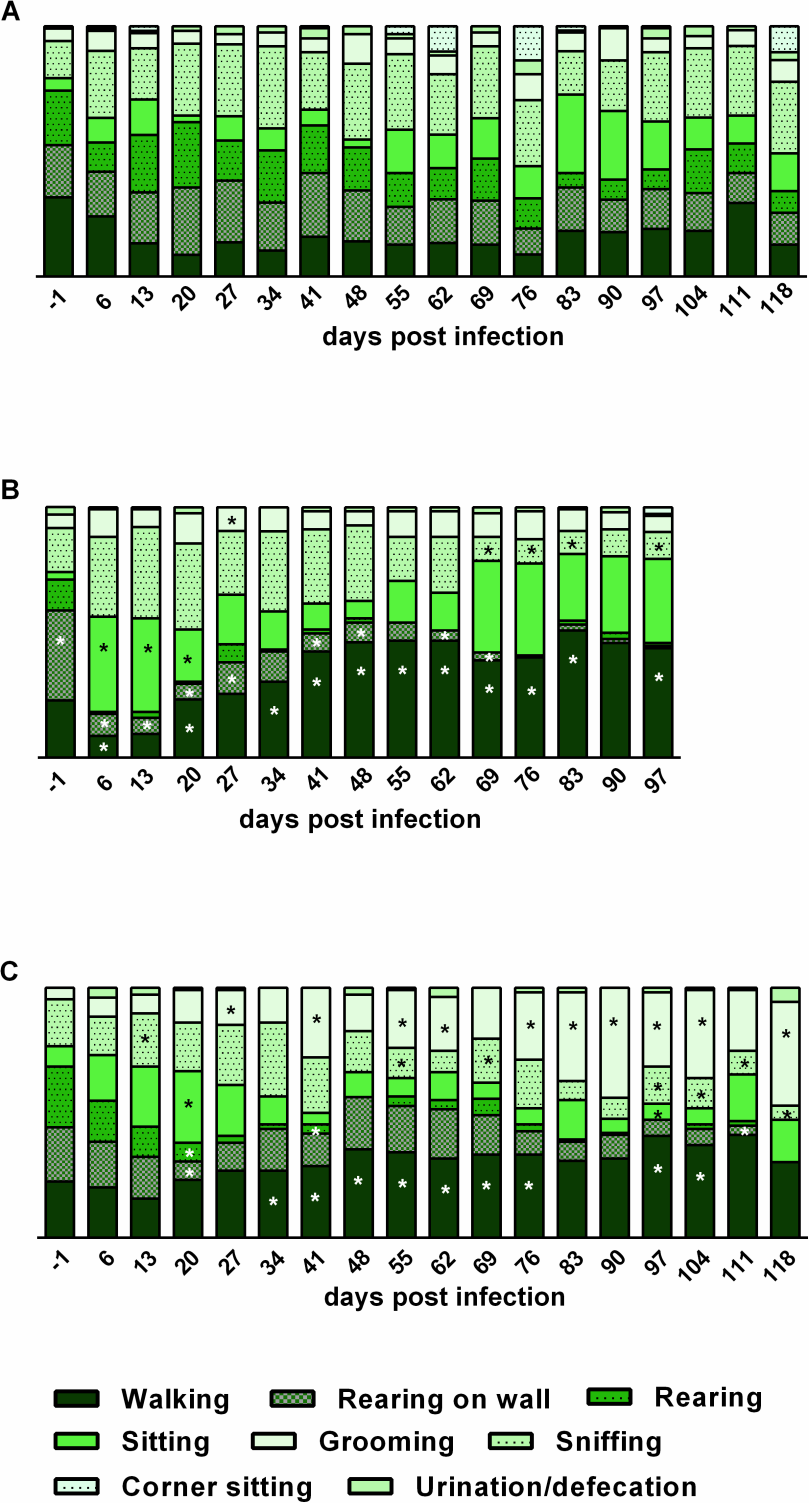
Alterations in activity patterns during the course of neurotoxocarosis. (A) uninfected control mice, (B) *T*. *canis*-infected mice, (C) *T*. *cati*-infected mice. Individual bars represent 100% of recorded activity with specific activities presented as respective fractions. Asterisks indicate significant differences in activities of the respective infection group compared to the uninfected controls. Note that intense reduction of “rearing on wall” and “rearing” in both infection groups did not allow presentation of asterisks in respective bars. Significantly reduced rearing on wall was additionally observed days 83–97 pi in *T*. *canis*-infected mice (B) and day 118 pi in *T*. *cati*-infected mice (C). Also, significantly reduced rearing was observed in *T*. *canis*-infected mice (B) days 6–20 pi and 34–97 pi days as well as in *T*. *cati*-infected mice (C) days 27–48 pi and 90–97 pi.

Both infection groups showed differences in activity patterns during the course of infection compared to the uninfected control. *T*. *canis*-infected mice revealed most severe differences to control mice mainly in walking activity, which was significantly more frequently observed starting day 34 pi. During the acute phase of infection from days 6–20 pi, *T*. *canis*-infected mice were also sitting more frequently than control mice. Rearing on cage walls was significantly reduced during almost the entire course of infection. In contrast, grooming was significantly more frequently observed in *T*. *cati*-infected mice compared to control mice starting day 27 pi, whereas *T*. *canis*-infected mice showed increased grooming behaviour solely day 27 pi. As similarly described for *T*. *canis*-infected mice, *T*. *cati*-infected mice were more frequently walking than uninfected controls starting day 34 pi until day 104 pi. Even though more frequent sitting was only observed day 20 pi, *T*. *cati*-infected mice were moving significantly slower than uninfected controls starting day 20 pi. In contrast, *T*. *canis*-infected mice were not significantly slower than uninfected controls until day 62. Rearing as well as sniffing was irregularly significantly reduced in both infection groups over the course of infection. Unusual behaviour such as spatial disorientation and stereotypical circling was exclusively observed in *T*. *canis*-infected mice, starting day 27 pi and 34 pi, respectively. Urination/defecation as well as corner sitting was rare (<5%) and no preference for any position within the cage was detected in all experimental groups. An overview of most commonly conducted activities is provided in Fig 5.

### Assessment of sensorimotor function

**Fig 6:**
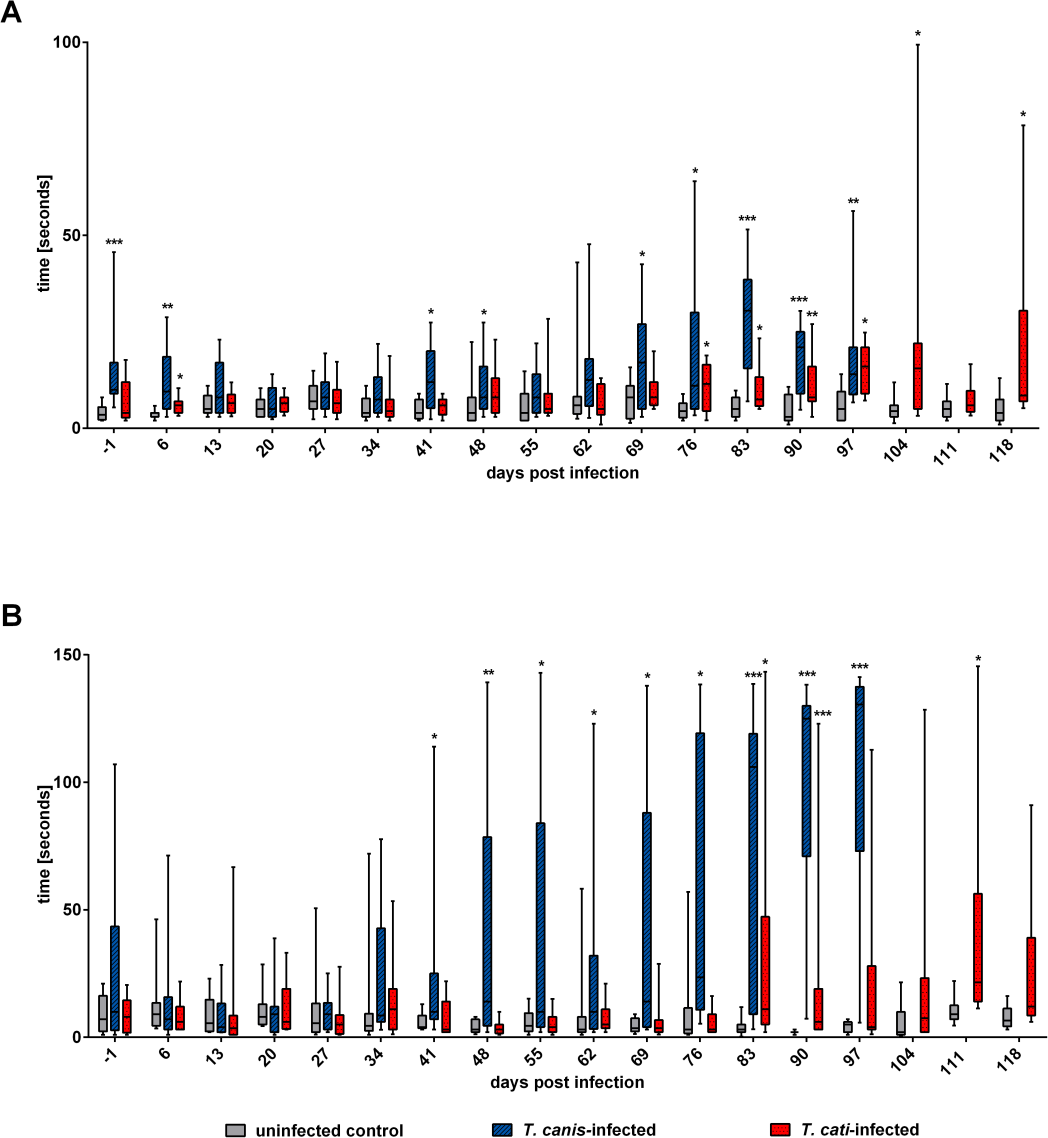
Assessment of sensorimotor function in *Toxocara canis-* and *T*. *cati*-infected mice. (A) Time-to-contact. Time required for mice to notice piece of tape on their front paws. (B) Time-to-remove. Time required for mice to remove piece of tape from their front paws. Ends of boxes define the 25th and 75th percentiles, with a line at the median and error bars defining the 10th and 90th percentiles. Asterisks indicate significant differences of the respective infection group compared to the uninfected control. *p≤0.05; **p≤0.001; ***p≤0.0001.

*Toxocara*-infected mice showed altered sensory as well as motor function over the course of experimental infection with *T*. *canis*-infected mice showing more impaired reactions than *T*. *cati*-infected mice. Initial significantly increased time-to-contact of infected mice may be attributed to unsatisfactory adaptation to respective handling prior infection. During progression of infection, *T*. *canis*-infected mice showed significantly increased values days 41 and 48 pi as well as the remaining five examination days 69–97 pi. Similarly, *T*. *cati*-infected mice showed significantly increased time-to-contact compared to control mice as of day 76–118 pi with exception of day 111 pi. Consistent significantly longer times for removing the piece of tape from their paw were observed days 41–97 pi for *T*. *canis*-infected mice. Contrary, *T*. *cati*-infected mice only showed significantly increased time-to-remove days 83 and 90 pi. Detailed information about time-to-contact and time-to-remove are provided in Fig 6.

### Assessment of memory function

**Fig 7:**
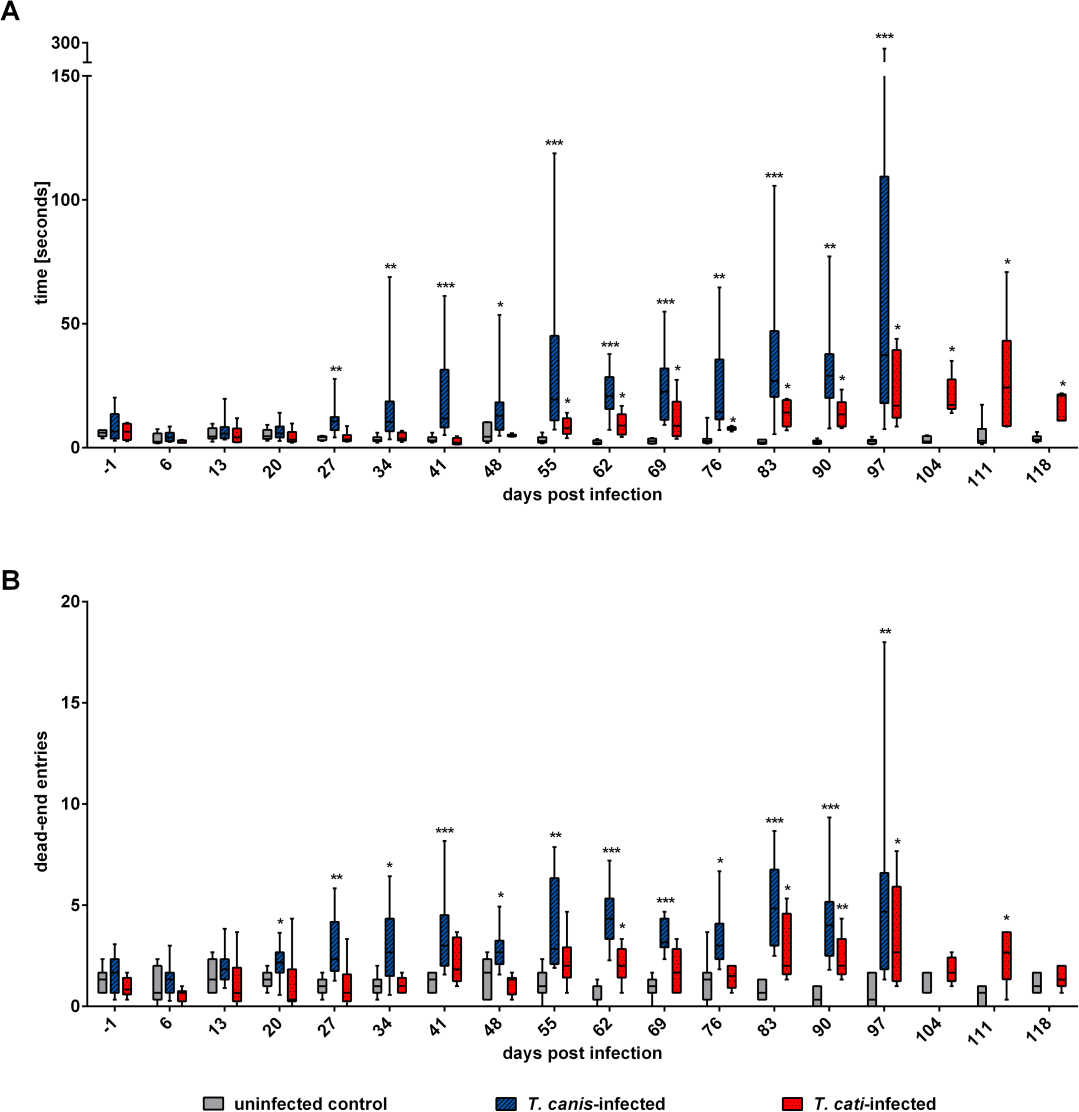
Assessment of memory functions in *Toxocara canis-* and *T*. *cati*-infected mice using the classic maze. A) Mean time required for finding food reward. B) Number of entries into dead-end arms. Ends of boxes define the 25th and 75th percentiles, with a line at the median and error bars defining the 10th and 90th percentiles. Asterisks indicate significant differences of the respective infection group compared to the uninfected control. *p≤0.05; **p≤0.001; ***p≤0.0001.

*T*. *canis*-infected mice required significantly more time to find the food reward than control mice starting day 27 pi. The mean duration upon finding the food reward increased over time, which was also observed regarding the number of entries into dead-end arms, starting to be significant as of day 20 pi. *T*. *cati*-infected mice required significantly more time than control mice 4 weeks later than *T*. *canis*-infected mice (starting day 55 pi), but did not show continuous entries into dead-end arms. Significantly more dead-end arms than control mice were solely entered on day 62 pi as well as days 83–97 pi. Detailed progression of impaired memory functions is provided in Fig 7.

## Discussion

*Toxocara*-induced NT may result in a variety of neurological symptoms such as behavioural alterations as well as neurological disturbances in paratenic hosts, including humans. To date, most studies are focusing on the influence of *T*. *canis* as the presumably more frequent causative agent of human toxocarosis. However, diagnosis remains challenging and the potential infection risk with *T*. *cati* larvae may be underestimated as it is generally considered high due to uncontrolled defecation habits of cats in public places like playground sandpits [29–31]. Nevertheless, studies regarding influence of *T*. *cati* larvae on behaviour and neurological functions as well as the direct comparison of *T*. *canis*- and *T*. *cati*-induced NT in terms of behavioural alterations and influence on memory function are rare. The presented study aids in filling the gap to provide a comprehensive picture of *T*. *canis*- and *T*. *cati*-induced NT and its progression in the mouse as model for human NT.

Both infection groups showed differences in general health and neurobehavioural parameters compared to uninfected mice, demonstrating the need to consider *T*. *cati* as a causative agent of human NT. Nevertheless, more severe pathogenesis in *T*. *canis*- than in *T*. *cati*-infected mice was observed. Alterations were mostly detected during the chronic phase of infection with effects of *T*. *cati*-infection appearing delayed compared to *T*. *canis*-infection. These phenotypic results considerably complement data regarding more severe progression in *T*. *canis*-infection, with concurrent delayed onset in *T*. *cati*-infection in terms of clinical symptoms as well as transcriptional and histopathological alterations in the brain [22,24,35,36]. In contrast to the presented study, previously conducted behavioural studies showed no differences in general health of *T*. *canis*-infected mice compared to uninfected controls, but behavioural differences were observed [37], possibly indicating adaptive manipulation. Differences to presented data may depend on different mouse strains as well as infection doses as these factors influence progression of infection [21,35,38,39]. These factors also demonstrate the importance of directly comparing effects of *T*. *canis*- and *T*. *cati*-induced NT under identical conditions to thouroughly characterize effects of the parasites on the host.

In *T*. *canis*-infected mice, most parameters associated with neurological dysfunction were deviant from behaviour of uninfected controls. Tumbling motions were well observed starting day 20 pi indicating an early onset of neurological involvement due to early migration of larvae to the brain. Less severe neurological dysfunctions in *T*. *cati*-infected mice may be attributed to generally lower larval numbers in the brain, even though early migration of *T*. *cati* larvae to the brain has also been observed [21,22,26]. Circling as stereotypical behaviour was solely detected in *T*. *canis*-infected mice supporting the observation of severe neurological involvement. Even though mentioned neurological dysfunctions result in a selective advantage regarding uptake by the definitive host, behavioural alterations are most likely attributed to side-effects of pathology—rather than adaptive manipulation—and may be coincidentally beneficial for the parasite [38,40]. This is also supported by increased excitability levels (indicated by response to a sudden sound stimulus) in *T*. *canis*-infected mice until day 41 pi, which is not in accordance with adaptive manipulation as this behaviour would prevent exposure of infected mice in the environment. By contrast, *T*. *cati*-infection mostly provoked reduced excitability as well as flight reaction when being picked up by the tail resulting in increased probability of ingestion by predators within the environment. In addition to a milder course of infection, neurological disturbances like tumbling motions were observed only sporadically leading to the assumption of a better adaptation of *T*. *cati* larvae to the paratenic host. Therefore, contrary to *T*. *canis*-induced NT, *T*. *cati*-induced behavioural alterations are likely to be attributed to adaptive manipulation to facilitate transmission of larvae. Reasons for this clearly demonstrated deviant influence of the two *Toxocara* species remain unclear. It may be speculated that lower larval numbers in addition to less severe pathology in *T*. *cati*-infected mice provokes the parasite to induce alternative strategies to facilitate transmission of larvae from the paratenic to the definitive host. Reduced defence during physical handling was most intensely observed in *T*. *cati*- and to a lesser extent in *T*. *canis*-infected mice. In *T*. *cati*-infected mice, this observation might be attributed to generally reduced flight behaviour; whereas reduced defence in *T*. *canis*-infected mice is most likely a result of described pathology. Reduced defence in combination with reduced inquisitiveness and exploration in both *Toxocara*-infected groups are in accordance with results of activity assessments in terms of reduced rearing and increased walking. Tendency of incuriosity about their environment as well as high activity levels (walking) in familiar or novel areas results in higher vulnerability to predators [41,42]; however, it remains unclear if this behaviour is attributed to pathology or adaptive manipulation. Even though suggested host manipulation is not beneficial for *Toxocara* spp. within the human host, pathology accompanied by behavioural as well as neurological alterations such as confusion, ataxia or dementia severely influences quality of life of affected patients [32]. Wether human *Toxocara*-infections result in personality changes as well as delayed reaction times, increasing the risk of e.g. accidents as described for human *Toxoplasma*-infection [43,44], has not been investigated yet.

Interestingly, the extensive grooming behaviour in *T*. *cati*-infected mice during activity assays was observed prior to ruffled, partially dirty, appearance during the chronic phase of infection (day 27 pi vs. day 62 pi). Ruffled appearance may be connected to effects of larval migration on general health as *T*. *cati*-infected mice also showed more severe deviations in body position compared to *T*. *canis*-infected mice, possibly indicating persistence of *T*. *cati*-larvae and pathological involvement throughout the skeletal muscle [22,35]. This persistence could also affect muscle function, nevertheless, *T*. *cati*-infected mice only showed sporadic inability to hold on to the vertical screen, whereas *T*. *canis*-infected mice showed marked inabilities during the course of infection, possibly attributed to reduced muscle strength [45,46] as well as poor general health. Furthermore, ruffled appearance of *T*. *cati*-infected mice and especially enhanced grooming may result from disturbances in hormone and neurotransmitter levels as these are inducing factors for altered self-grooming behaviour in rodents [47–49]. Self-grooming has therefore been discussed as an indicator for repetitive behaviour and consequently as potential model for human psychiatric disorders [48]. Disturbed levels of neurotransmitters e.g. depressed levels of dopamine are implicated in behavioural alterations in a variety of psychiatric diseases like schizophrenia and depression [50,51]. A potential correlation between *Toxocara-*seropositivity and neuropsychiatric disorders like schizophrenia, seizures and cognitive deficits in human patients has been discussed [32]. Reduced dopamine as well as serotonin and GABA levels in *T*. *canis*-infected mouse brains [50] highlight the need for a more detailed characterization of NT to confirm potential correlation. As a variety of neurotransmitters induce a reduction of rearing behaviour and also modulate stress- and anxiety-related behaviour in rodents [48,49], recorded behavioural alterations may also be attributed to respective dysregulations. However, contrary to *T*. *canis*-infection, altered neurotransmitter levels during *T*. *cati*-induced NT have not been investigated yet. It may be speculated that *T*. *cati*-infection induces a different pattern of neurotransmitter dysregulation, which may account for observed marked increased grooming and reduced flight behaviour. Different neurotransmitter dysregulations may therefore be hypothesised as a key factor for differences in behavioural alterations during *T*. *canis*- and *T*. *cati*-induced NT.

Sensorimotor assessment indicated reduced sensation on paws, which was also observed earlier in *T*. *canis*- than in *T*. *cati*-infected mice with motoric impairment being more extensive in *T*. *canis*-infected mice. As some of the core functions of the cerebellum are coordination and balance, structural damage influences those functions. Recorded motor incoordination as well as ataxia may therefore be assigned to the pathological alterations in cerebellar tissue observed in previous studies [21,52,53]. Less severe structural damage during *T*. *cati*- in comparison to *T*. *canis*-induced NT [22] may contribute to less severe impairment in *T*. *cati*-infected mice. The altered sensorimotor behaviour in both infection groups may particularly be attributed to occurring axonal damage which is associated with neuromotor and sensory dysfunction [22,24,54]. Motor impairment as well as sensory impairment and dysesthesia in *Toxocara*-positive humans [12,55–57] may therefore also be attributed to axonal damage, particularly requiring further investigations. As mice predominantly sense the strip on their paw even if delayed, reduced removal may be an effect of impaired balance of *T*. *canis*-infected mice. In contrast, prolonged removal times of *T*. *cati*-infected mice may not result from motoric impairment in terms of balance, but rather the neurological phenomenon “slower motion” already recorded during activity assays. The general slower motion in *T*. *cati*-infected mice also contributes most likely to significantly longer duration in the maze before finding the food reward compared to uninfected controls. Nevertheless, maze results show a significant effect on memory function in both infection groups. To our knowledge, this is the first report on *T*. *cati* larvae influencing memory function of the paratenic host. Again, *T*. *canis*-infected mice showed an earlier onset of impaired memory function as several attempts to fulfil the task starting day 20 pi were required. *T*. *cati*-infection had a less pronounced effect on memory and condition may have been transient as infected mice were not consistently requiring more dead-end entries than uninfected controls during the chronic NT phase as was the case with *T*. *canis*-infected mice. Transient behavioural alterations have been described in *Toxocara*- as well as in *Toxoplasma*-infected rodents, nevertheless, strains and infection doses were not comparable to the present study, which is an influential factor in disease progression [21,35,46,58]. Less pronounced alterations in *T*. *cati*-infected mice may be on account of fewer larvae in the brain, as *Toxocara* larval or *Toxoplasma* cysts numbers correlate with the degree of behavioural alteration [22,26,59]. Memory impairment in rodents may strongly influence survival factors like recalling resources and use of visual cues. As in the presented study, similar experiments provided evidence of memory impairment as *T*. *canis*-infected mice required significantly longer times than uninfected controls to locate vital resources like water in a given area after a preceded deprivation period [26]. Cerebellar lesions and damage in *Toxocara*-infected mice may contribute to impairments as parts of the cerebral cortex involved in cognitive regulation are connected to the cerebellum. Therefore, cerebellar integrity is required for cognitive functions [54] and may be influenced by the infection. Suspected neurodegeneration in *Toxocara*-infected mice [28] may particularly influence observed memory dysfunction as neurodegenerative cerebellar diseases in humans have been shown to influence explicit memory without affecting implicit memory [24,32,52]. Therefore, results obtained in mice may be comparable to observed memory impairment of *Toxocara*-positive human patients [7,10,11,13] as explicit memory, an intentional process to recall facts and experiences, is required to recall the location of the food reward. It may therefore be speculated that neurodegeneration constitutes an important part in human NT. Additionally, particular brain regions may be affected by larval persistence and adverse immune reactions. Damage of e.g. the hippocampus, which is implicated in development and maintenance of spatial learning as well as reduced exploratory behaviour [54], may result in recorded alterations. However, a detailed characterization of brain areas involved during NT is not yet available. Such investigations are desirable to better comprehend behavioural or neurological alterations in the infected host. Correlation of learning impairment and *Toxocara*-seropositivity in children additionally highlights the need for further detailed characterization of NT [15,17].

The current results clearly show that *T*. *cati*-induced NT also influences behavioural parameters, even though to a lesser extent than *T*. *canis*-induced NT. It still may be assumed that neurological disturbances such as ataxia and balance incoordination in humans may mostly be attributed to the—extrapolated from the mouse model—likely more severe *T*. *canis*-infection. However, based on obtained results, memory impairment as well as cognitive dysfunction frequently observed in NT patients [7,10,11] may be induced by either *Toxocara* species. *T*. *cati* should therefore strongly be considered as a causative agent of human NT as well. Overall, the conducted study evidently demonstrates an earlier onset of severe neurological and behavioural alterations in *T*. *canis*-infected mice with more severe progression of disease than in *T*. *cati*-infected mice. Observed differences in behavioural and neurological alterations provide valuable hints towards varying key regulatory mechanisms during *T*. *canis-* and *T*. *cati*-induced NT, contributing to a broader understanding of NT, which will aid in determining targets for prevention as well as novel diagnostic and therapeutic approaches.

## Supporting information

S1 Appendix Physiological status and neurobehavioural phenotyping. Parameters and scoring modified according to Irwin (1968).
(DOCX)

S2 Appendix Assessment of general activity. Parameters and scoring modified according to Irwin (1968).
(DOCX)

S1 Fig Classic maze for assessment of memory function. Schematic overview of the maze used in the presented study. The star indicates the starting position of mice and the flag the position of the food reward.
(TIF)

S2 Fig Mean body weights and temperatures of uninfected controls and *Toxocara*-infected mice during the course of infection. A) Body weight and B) body temperature. Ends of boxes define the 25th and 75th percentiles, with a line at the median and error bars defining the 10th and 90th percentiles. Asterisks indicate significant differences of the respective infection group compared to the uninfected control.
(TIF)
