# Supplementary material for: Abnormal neurobehaviour and impaired memory function as a consequence of Toxocara canis- as well as Toxocara cati-induced neurotoxocarosis
Source: PLoS Negl Trop Dis. 2017 May 8;11(5):e0005594. doi: 10.1371/journal.pntd.0005594 (PMC5436879; doi:10.1371/journal.pntd.0005594)
Supplement: S1 Appendix — Parameters and scoring modified according to Irwin (1968). (DOCX) [file pntd.0005594.s001.docx]

**S1 Appendix: Physiological status and neurobehavioral phenotyping -** parameters and scoring modified according to Irwin (1968).

**1. Reaction during tail fixation**

0 – no flight reaction

2 – moderate flight reaction

3 – strong flight reaction

4 – jumping/aggressiveness

**2. Hind limb tension during transfer**

0 – no extension of hind limbs

1 – reduced extension of hind limbs

2 – normal extension of hind limbs

**3. Arousal upon transfer**

1 – prolonged freezing (more than 5 seconds)

2 – short freezing with subsequent movement

3 – immediate movement

4 – extreme excitement, manic

**4. Body position**

0 – flat in abdominal position

1 – lateral position

2 – normal body position

3 – bend body position

4 – sitting body position

5 – tense/cramped body position

**5. Tail elevation**

0 – no elevation, dragging

2 – normal elevation

3 – 45° tail elevation

4 – 90° tail elevation

5 – tail elevated above body

**6. Respiration**

0 – gasping, acute dyspnoea

1 – reduced respiratory rate

2 – normal respiratory rate

3 – elevated respiratory rate

**7. Skin colour/circulation**

0 – cyanosis (skin bluish)

1 – whitish skin, vessels barely visible

2 – slightly pink skin

3 – dark pink skin

4 – red skin

**8. Coat appearance**

2 – clean

3 – ruffled

4 – partially dirty

5 – dirty

**9. Exophthalmos**

2 – no

3 – yes

**10. Ptosis**

2 – eyelids normally open

3 – eyelids partially open (1/2)

4 – eyelids partially open (1/4)

5 – eyelids closed

**11. Lacrimation**

2 – no

3 – yes

**12. Salivation**

2 – no

3 – yes

**13. Response to sudden sound stimulus as indicator for anxiety**

0 – no shock reaction

1 – reduced, delayed shock reaction

2 – normal shock reaction, brief freezing

3 – massive shock reaction, flight, jumping, prolonged freezing

**14. Hearing (based on anxiety)**

0 – deaf

2 – able to hear

**15. Pelvic position**

1 – flattened/pushed towards ground

2 – normal (about 3 mm above ground)

3 – elevated (more than 3 mm above ground)

**16. Position of legs (ataxia)**

0 – massive adduction of legs

1 – slight adduction of legs

2 – normal leg position

3 – slight abduction of legs

4 – massive abduction of legs

5 – massive abduction of legs, no movement possible

**17. Tumbling motion**

2 – normal gait, no tumbling motion

3 – slight tumbling motion

4 – tumbling motion, falling to lateral position

5 – falling from one body side to the other when trying to move

6 – lateral position without getting up

**2. Handling**

**18. Balance** (slight movement of examination cage)

0 – reduced

2 – normal (mouse maintains balance)

**19. Inquisitiveness** (presentation of object, e.g. pen, about 2 cm frontal of mouse)

0 – no reaction

1 – reduced (solely movement of head)

2 – normal: contact with vibrissae (movement of entire body)

3 – increased: contact with nose

4 – aggressiveness (attacking of object)

**20. Touch-escape** (stroke with brush on mouse‘ back)

0 – no reaction

1 – slow evasive movement

2 – moderate evasive movement

3 – rapid evasive movement

5 – vehement escape/aggressiveness as response to even light touch

**21. Vibrissae reflex** (touching vibrissae with brush)

0 – no reaction

2 – normal reaction

3 – movement of entire head

4 – movement of entire body

**22. Exploration behaviour** (centred positioning of mice on platform, allow to explore for a maximum of 15 seconds)

0 – no reaction (mouse remains in centre position)

1 – no exploration of platform edges

2 – exploration of entire platform

3 – exploration and jumping off platform

4 – jumping off platform without exploration

**23. Postural reflex** (placing of mouse on screen)

0 – no reaction

2 – extension of front limbs

**24. Forelimb placing reflex** (in combination with postural reflex)

0 – no reaction

2 – gripping of screen bars

**25. Vertical screen test** (turning screen horizontal to vertical within 2-3 seconds)

2 – mouse shows typical posture, gripped screen bars upon contact and was able to hold on for at least 5 seconds on vertical screen or climbed to upper edge of screen

1 – typical posture questionable, weak grip to screen bars and weak hold, no climbing to upper edge of screen, panic reaction in terms of uncontrolled jumping

0 – typical posture questionable, no grip to screen bars, no persistence on vertical screen

**26. Righting reflex**

2 – no impairment

1 – delayed straightening (up to 2 seconds)

0 – massively delayed straightening (more than 2 seconds)

-1 – no straightening from dorsal position

**27. General handling behaviour**

1 – defenceless

2 – moderate resistance

3 – aggressive, strong flight reaction
