# Supplementary material for: Abnormal neurobehaviour and impaired memory function as a consequence of Toxocara canis- as well as Toxocara cati-induced neurotoxocarosis
Source: PLoS Negl Trop Dis. 2017 May 8;11(5):e0005594. doi: 10.1371/journal.pntd.0005594 (PMC5436879; doi:10.1371/journal.pntd.0005594)
Supplement: S2 Appendix — Parameters and scoring modified according to Irwin (1968). (DOCX) [file pntd.0005594.s002.docx]

**S2 Appendix: Assessment of general activity** - parameters and scoring modified according to Irwin (1968).

**1. Speed of movement**

0 – immobile

1 – slow

2 – normal

3 – rapid

**2. Activities**

**W** – walking

**G** – grooming

**S** – sitting

**CS** – sitting in corner

**SN** – sniffing

**R** – rearing

**RW** – rearing against the wall

**UF** – urinating/defecation

**3. Abnormal neurological behaviour**

**HF** – head flicking or head shaking

**HS** – head searching (repetitive)

**H** – “hallucinating” - mouse appears to respond to objects not present (possibly “boxing”)

**B** – compulsive biting

**L** – compulsive licking

**SB** – self destructive biting - usually of toes with bleeding

**P** – prancing forelimbs - shifting from one forelimb to another

**UW** – upright walking

**AW** – aimless wandering

**Ci** – circling

**WA** – waltzing - rapid turning in circles

**R** – retropulsion - animal walks backwards

**D** – spatial disorientation - walking or stumbling into wall

**HB** – head bobbing

**SG** – stop&go, animal walks a few steps, stops and walks again

**4. Seizures**

**Clonic type: alternative contraction and relaxation of the voluntary muscles**

**C** – clonic - coordinated, unsymmetrical convulsion and natural, purposeful like movements, e.g. running

**Csy** – clonic symmetrical - repetitive symmetrical jerks or twitches of the limbs

**Rn** – running excitement - often accompanied by mild clonus or leading to severe convulsion

**Ch** – champing - clonus of jaws only

**P** – popcorn - seizure with animal repeatedly “popping” into the air

**A** – asphyxia - terminal clonic or clonic-tonic convulsion resulting from respiratory failure

**Tonic type: persistent contraction and spasm of a set of voluntary muscles**

**T** – tonic sustained extension of hind limbs, usually preceded by tonic flexion

**Op** – opisthotonus - head, body and limbs are rigidly extended and arched backwards

**Em** – emprosthonus - opposite of Op, e.g. extended

**Rr** – rock & roll - mouse is prostrated on its back and rocks from side to side, trying to right itself

**Su** – sitting up - mouse sits upright on hind limbs during seizure

**Pr** – praying - mouse is sitting upright and forelimbs are held together or crossed in attitude resembling prayer
