# Supplementary figures and images for: Abnormal neurobehaviour and impaired memory function as a consequence of Toxocara canis- as well as Toxocara cati-induced neurotoxocarosis
Source: PLoS Negl Trop Dis. 2017 May 8;11(5):e0005594. doi: 10.1371/journal.pntd.0005594 (PMC5436879; doi:10.1371/journal.pntd.0005594)

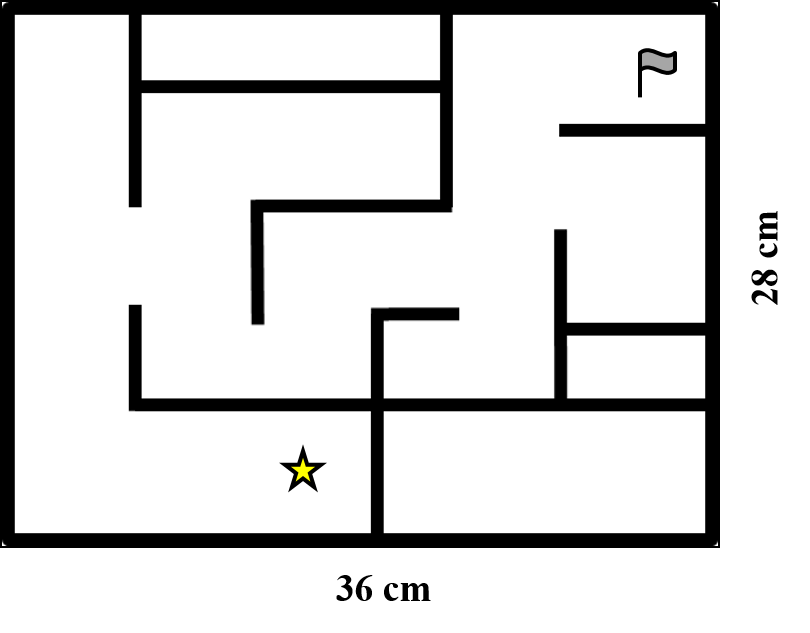

Supplement: S1 Fig — Schematic overview of the maze used in the presented study. The star indicates the starting position of mice and the flag the position of the food reward. (TIF) [file pntd.0005594.s003.tif]
